# Performance Analysis of Indentation Punch on High Energy Lithium Pouch Cells and Simulated Model Improvement

**DOI:** 10.3390/polym13121971

**Published:** 2021-06-15

**Authors:** Lihua Ye, Muhammad Muzamal Ashfaq, Aiping Shi, Syyed Adnan Raheel Shah, Yefan Shi

**Affiliations:** 1 School of Automotive and Traffic Engineering, Jiangsu University, Zhenjiang 212013, China; 2 School of Agricultural Equipment Engineering, Jiangsu University, Zhenjiang 212013, China; shap@ujs.edu.cn; 3 Department of Civil Engineering, Pakistan Institute of Engineering and Technology, Multan 66000, Pakistan; syyed.adnanraheelshah@uhasselt.be; 4 Department of Electrical and Computer Engineering, Binghamton University, Binghamton, NY 13902, USA; yshi37@binghamton.edu

**Keywords:** lithium-ion, high energy pouch cell, state of charge, electrolyte, load position, modeling

## Abstract

In this research, the aim relates to the material characterization of high-energy lithium-ion pouch cells. The development of appropriate model cell behavior is intended to simulate two scenarios: the first is mechanical deformation during a crash and the second is an internal short circuit in lithium-ion cells during the actual effect scenarios. The punch test has been used as a benchmark to analyze the effects of different state of charge conditions on high-energy lithium-ion battery cells. This article explores the impact of three separate factors on the outcomes of mechanical punch indentation experiments. The first parameter analyzed was the degree of prediction brought about by experiments on high-energy cells with two different states of charge (greater and lesser), with four different sizes of indentation punch, from the cell’s reaction during the indentation effects on electrolyte. Second, the results of the loading position, middle versus side, are measured at quasi-static speeds. The third parameter was the effect on an electrolyte with a different state of charge. The repeatability of the experiments on punch loading was the last test function analyzed. The test results of a greater than 10% state of charge and less than 10% state of charge were compared to further refine and validate this modeling method. The different loading scenarios analyzed in this study also showed great predictability in the load-displacement reaction and the onset short circuit. A theoretical model of the cell was modified for use in comprehensive mechanical deformation. The overall conclusion found that the loading initiating the cell’s electrical short circuit is not instantaneously instigated and it is subsequently used to process the development of a precise and practical computational model that will reduce the chances of the internal short course during the crash.

## 1. Introduction

In recent decades, energy derived from fossil fuels has become harmful to the climate and its sources are decreasing with time [1]; that is why electric vehicles are expected to replace vehicles in which gasoline is used as an energy source. To overcome that deprivation of this energy in the future, batteries are considered to be one of the significant methods of storing energy that is being produced by different sources. Lithium-ion batteries have proven to be very profitable, and they are used in many electronic gadgets [2]. Moreover, they are also not used in various electric sources, such as mobile phones, laptops, and cameras, but are widely used as energy storage systems in electric vehicles due to their high capacity, low self-discharge, long life, high energy density, and low climatic effect characteristics. In some conditions, lithium batteries have also proven to be the cause of thermal runaway, mechanical abuse, and release of toxic gases [2,3,4,5]. Thus, interest in lithium-ion batteries from researchers has increased in recent years [6,7,8,9,10,11]. Electrochemical technologies are particularly significant in present society, and it is an exciting time for researchers that are active in this sector [12]. This is a vital topic for research for electric vehicles, specifically when considering the massive operational range that electric vehicles would be subjected to [13,14,15].

Sony Ericson established the first battery in 1991, and a lithium-ion battery was later commercially introduced [16,17]. Since then, millions of cells are in the market with their different specifications [18,19,20]. For many years, customer’s demand and best purposes have persisted in maintaining more extended battery running time. To meet customer’s needs, producers have appropriately responded to increase battery performance by changing the cell’s chemistry, which directly affects its memory loss and high electric density. Different li-ion cells include various types of materials, and the material containing titanium [21,22,23], silicon, and graphite oxide for the negative electrode, LiMOy, in which oxides, manganese oxide (MOy), cobalt oxide, and ferrous phosphate, etc. can be used as a metal oxide [24]. Direct contact between electrodes can cause a short circuit. A separator is used for this type of short circuit to make a cell safe [25,26,27]. A separator is made of different materials, such as polypropylene, for a lithium polymer cell. Still, in the lithium-ion cell, SEI (Solid Electrolyte Interphase) usually consists of Li_2_O (Lithium Oxide) [28,29], LiF (Lithium Fluoride) [30], Li_2_CO_3_ (Lithium Carbonate) [31], and polyolefin [32,33,34]. The electrolyte depicted in this study consists of a conductive salt, e.g., lithium hexafluorophosphate (LiPF6), and a solvent dimethyl carbonate (DMC), and ethylene carbonate (EC), diethyl carbonate (EC), or ethyl methyl carbonate (EMC) [35]. The use of metallic lithium leads to safety hazards [36] and low cycle efficiency due to lithium’s reactivity with ordinary liquid electrolytes [37]. The energy density of a lithium-ion battery is higher than other battery sources. In the pouch/prismatic cells, one thing that is more common is that the electrodes/separator assembly is not wrapped in its mount. The length to thickness ratio, rather than the actual dimensions, is the thing that matters; the prismatic battery is regarded as an-isotropic multi-layered thick plate [38,39,40].

There is a need to overcome these internal short circuits due to mechanical failure which is due to accidents, according to the International Energy Agency [40,41,42,43]. Unfortunately, manufacturers could not provide the reasons and mechanisms of internal structural failure. Detailed computational modeling of the battery can explain the origins of failure, mechanisms of failure, and their implications to the battery pack design; thus, it can provide suitable enhancements in format [44]. Some previous studies are improving their mechanical and electrical performance by changing their material structures [45]. As a result, this led to battery-pack designs in EV (Electric Vehicle). Battery protection was examined [46]. Researchers observed changes by applying different loading conditions on lithium-ion battery cells, and they investigated the effects on cells while using the load in various conditions [47,48,49]. Suppose that an accident or mishap occurs in an electric vehicle. In that case, it can also harm the battery pack by intruding on external objects, which can lead to the mechanical deformation of lithium-ion cells. These external objects can occur from various directions, e.g., in-plane loading, which can compress the cell’s narrow border, and out-of-plane indentation, which can punch a vast surface of pouch cell perpendicularly [50,51,52]. Electricity, thermal, and mechanical integrities are the three main factors that are mutually related to battery protection [53,54]. Crash experiments of an EV provide a lesson from where it was once found that, instead of minor intrusion into a battery pack, a disastrous result was produced [55,56,57]. However, its overcharging also includes one of the most severe safety issues for applications of lithium-ion batteries [30,58]. Analysis of outcomes from quasi-static loading states on the reaction of the cell and most of the primary research and modeling work were done [58,59,60].

Several quasi-static indentation tests were carried out on lithium-ion pouch cells. These indentation tests were prompted at the onset of the internal short circuit. The location of fracture occurs before catastrophic failure of the entire pouch cell with unusual local damage, and accretion appears precisely on the layers. The transient breach occurs in the higher portion of the covered layers, the main force drops, and the synchronous strike of an inner short circuit occurs within the sac cell, while the down part of the pouch cell comprehensively stays. During the indentation tests process, for lithium pouch cells, the common structural unyielding increases at first before its final drop, and it then enters a stagnant condition. After the curvature point, a load of indentation makes the viscid forces stronger. It aggravates the separator into constituent layers and the particles of graphite de-coat from the anode [61]. The overall performance of the lithium-ion battery can be understood when researchers also investigated the status of mechanical stress on a lithium-ion battery, which is mainly caused by external stress. A variety of different mechanical models have been established to investigate the effects on lithium-ion battery packs by applying mechanical loads, which is one of the prominent reasons for the internal short circuit [62,63]. A collective numerical, analytical, and experimental approach was performed to develop a new cell failure model and to understand the primary mechanism of failure. As soon as the large format pouch cells were subjected to indentation leading to failure, an examination of failure regions demonstrated a fracture surface angle to the battery plane [64,65]. The research combined both experimental and computational studies [66,67]. Dynamic finite element simulations were performed to study pouch cells’ mechanical behavior, such as internal interfacial behavior, loading, and boundary conditions, which shows a direct interaction between cell boundaries and impactor leading to the significant change in the residual velocity [68,69].

**Figure 1:**
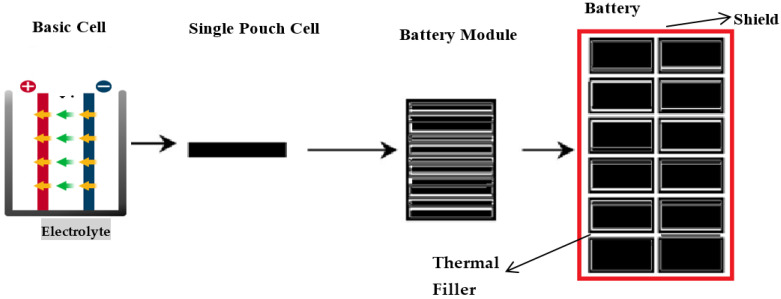
Preferable sketch of lithium-ion battery pack design and outer shield.

This research will focus on complete cell indentation tests on a specific model of high-energy pouch lithium-ion cells to facilitate the exploration of buckling response under various confinement levels. It also focuses on determining the effects on electrolytes while applying a different type of load by changing the punch size and punch position with the other soc. It will help to identify a model for battery cells when different mechanical loads are applied to it. Taking some measures can improve the application of lithium-ion batteries in different technologies. These measures can include strengthening the batteries’ walls, the storage chambers, material strength, compartment ventilation for fire suppression, and ingress and egress points. This indicates that cell battery model will help to build blocks for developing a battery pack model, as shown in Figure 1. It is a homogenized form of the battery cell where all five distinctive materials are smudged into one homogenized medium in the battery pack. Theoretically, even though provisional imperfections or modal patterns may be added, such a homogenized model will not simulate realistic buckling patterns because the battery’s actual configuration is anisotropic.

The comprehensive computational modeling will offer valuable information in the allocation to close the design feedback loop, leading to a robust design overall and providing the best solution.

## 2. Materials and Methods

### 2.1. Mechanical Indentation

The mechanical indentation test has been used in this study, including information regarding the physical and mechanical properties, the electrochemical characteristics, and the operating properties of the cells. Manufacturers are requested to discharge the cell of no more than 10% state of charge to avoid extreme reactions at the point of indentation tests. The other two cells have more than 10% state of charge with preventive measures to maintain the environment from severe reactions.

These types of cells are typical of those that can be found in electric vehicle applications or an energy storage system due to their incredible efficiency and high capacity [38,39]. These cells also have a stackable geometry and packaging. Lithium-ion cells provide compelling advantages to manufacturers. Any desired shape can be produced according to the requirement of the customer. Space and weight requirements can be met for mobile devices and notebook computers [70,71]. Testing on four pouch-type cells of li-ion is conducted in this work. The following sections describe the equipment, preparation, and results from those tests. Firstly, the Punch Indentation testing results are described after the battery component descriptions.

**Figure 2:**
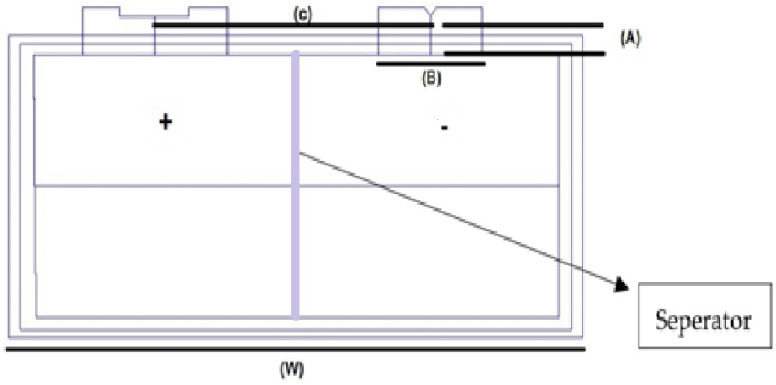
High-energy lithium pouch cell diagram.

In this study, High-energy lithium-ion pouch cells GPLFP (Gee Power LiFe Polymer Battery (Cell Manufacturing Company Guangdong, China) 11192320ES-50 Ah have been used, as depicted in Figure 2. Lithium-ion accumulators have 95% high discharge efficiency, which also shows low self-emit when compared to rechargeable cells, such nickel-cadmium and nickel manganese accumulators. There is an energy density of more than 200 W-h/kg for the commercially available lithium-ion battery.

Lithium presents advantageous properties, such as greater battery life, being present in many weights, reliability, foolproof safety, more energy repository, balance, and compatibility [18]. Figure 2 shows the major specifications of this cell. The online information sheet for this cell is provided in [72] to the interested reader and it is also shown in Table 1. Lithium pouch cells consist of stacked layers of anodes, separators, and cathodes that are sandwiched between the laminated film layers. These pouch cells can be created in customized sizes and shapes. Pouch cells are then linked in sequence and parallel to accumulate the preferred voltage and capacity. In a pouch cell configuration, “S” in the number specifies how many are in the series, and the “P” specifies the number of pack assemblies. For example, if you have a 4s4p pack, this would be a total of sixteen cells—four packs of four batteries each.

### 2.2. Indentation Test

**Figure 3:**
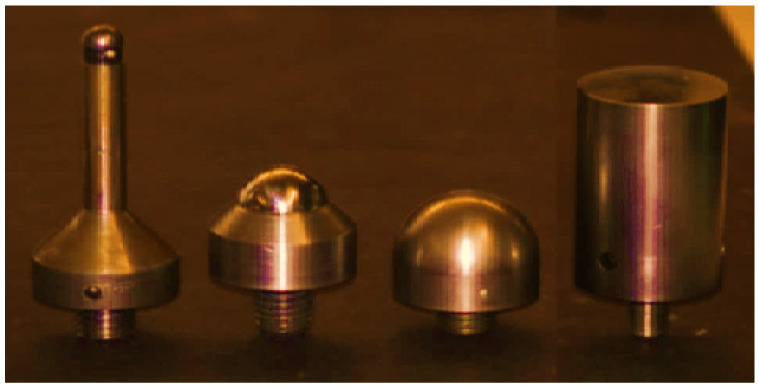
Indentation, different sizes of punch are used, which are hemispherical punches of diameters 12 mm, 28 mm, and 44 mm, and a flat cylindrical punch of 25 mm.

**Figure 4:**
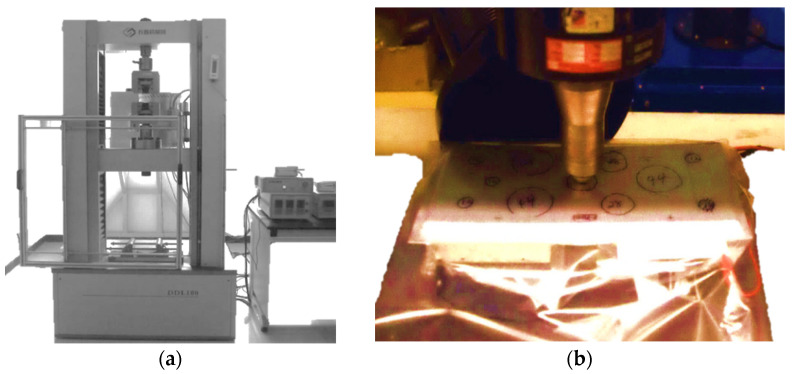
(**a**) Punching on the cell during the test (number of punches with different size were used) (**b**) Universal testing machine >50 kN.

Before testing, the cells have been discharged to the preferred discharge cut-off voltage of 2.5 V [72] to stop intense reactions all through the testing scenarios. Two cells have been on more than 10% soc and two cells have been kept on less than 10% soc. A complete test application has been performed to complete the calibration process and validate the corresponding models. Figure 3 presents four types of punch used in experiments that were carried out on the lithium-ion battery. Table 2 represents total number of punch tests applied on each cell. The cells were tested using the universal testing machine (UTM) loading frame that was equipped with a 200 kN load cell in the whole experiment, as shown in Figure 4. The first test of each cell included voltage monitoring in determining the onset of the internal electric short circuit. The punches have been placed in the center and on edges of the cell lengthwise and widthwise. All of these punches are used for all four cells. No punch is specified for any specified cell. Each cell used to be subjected to 10 consecutive indentation tests using three sizes of hemispherical punches-small (12 mm diameter), medium (28 mm diameter), and massive (44 mm diameter), and a flat cylindrical punch (25 mm diameter) [9,72], as can be seen in Figure 3.

### 2.3. Testing Procedure of Punch Indentation

A complete test application has been performed to complete the calibration process and validate the corresponding model. In preparation for testing, the cells have been separately placed in two-gallon Ziploc bags with SOLUSORB^®^ solvent adsorbent to include any electrolyte leakage that can also occur throughout the testing procedure. The bags have been then marked to determine appropriately spaced punch locations, such that the preceding tests would no longer influence the subsequent identical cell indentation test results. The prepared cell was then positioned on a stable, flat metallic surface under the loading frame with the suitable test location being found below the hemispherical punch to be used. For the first test of every cell, voltmeter leads have been linked to positive and negative cell terminals inside the bag using alligator clips, and the container used to be semi-sealed around the edges. The punch used is lowered to a point entirely above the cell’s surface. A fume extractor was placed inside six inches of the setup to remove the poisonous fumes released during and post-test. Figure 4 presents a standard, first-run test setup. The voltmeter was removed in subsequent identical cell test runs.

The following parameters are measured at 1-s intervals using meter view c software:

- The test computing device and displacement over time. In all of these tests, displacement is constant with a fixed rate of 1 mm/min., while related testworks04 software measured and recorded test machine load and crosshead displacement in 1-s intervals.
- Over time, cell voltage has been determined with a standard voltmeter at the end of the cell positive and negative electrodes. Voltage measurements have been measured simultaneously on the first test of each cell using a general voltmeter that is connected to the cell terminals and have been recorded at 1-s intervals using meter view c software.
- The outside temperature of a cell over time, as determined by thermo-couple.
- It is also mentioned that all of these tests are carried out at room temperature because, under intense cold or warm temperatures, the material properties can appreciably change, and the min-max range of temperature for batteries (−20 °C to 60 °C) is essential at a variety of variable temperatures above the characterization and evaluation be carried out.
- Photographs during tests are captured at a rate of average 5 min.
- When one short circuit is achieved, all of these monitoring devices work until these parameters again come back to their constant state.

Many tests are conducted to achieve fast reduction in force to locate punch on cells and the voltage readings indicating short circuit of cell. Between identical cell test scenarios, the loading frame used to be lifted and reset for cell repositioning and punches change-out when necessary.

## 3. Results

### 3.1. Testing Results of Punch Indentation

**Figure 5:**
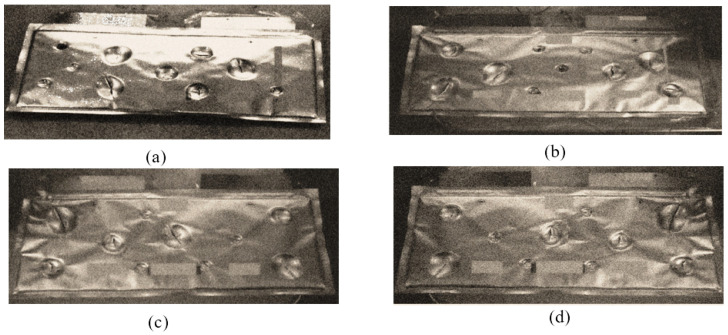
Cell-1 post test (**a**), cell-2 post test (**b**), cell-3 post test (**c**), and cell-4 post test (**d**).

Observing previous cells’ tests revealed no leakage or pretension of the pouch cells due to short circuit chemical reactions, and there will be no gas formation because it can cause an explosion. Nevertheless, a, b, c, d reveal the observational test results following the completion of all testing, as shown in Figure 5.

**Figure 6:**
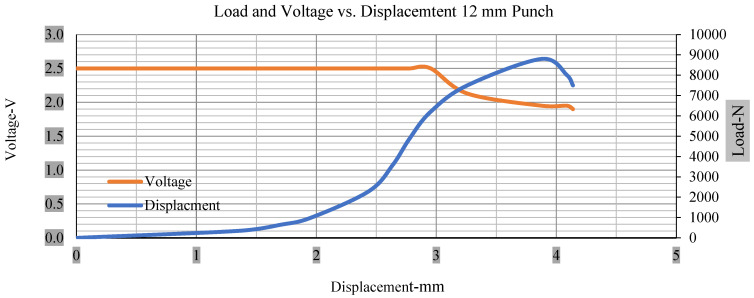
Load and voltage vs. displacement 12 mm punch greater than 10% soc.

**Figure 7:**
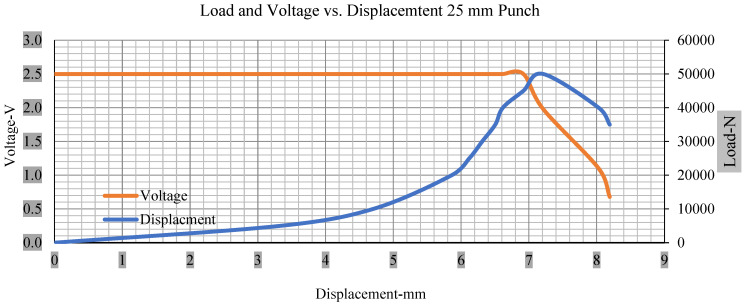
Load and voltage vs. displacement 25 mm punch greater than 10% soc.

**Figure 8:**
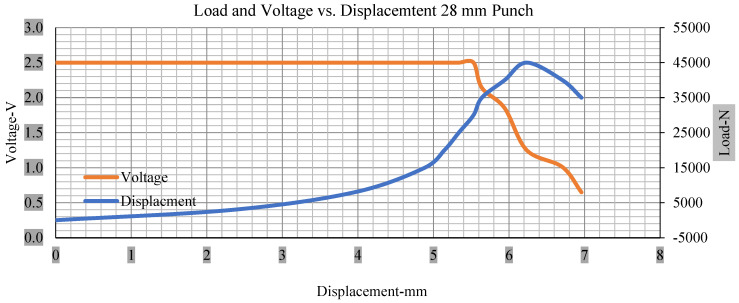
Load and voltage vs. displacement 28 mm punch greater than 10% soc.

**Figure 9:**
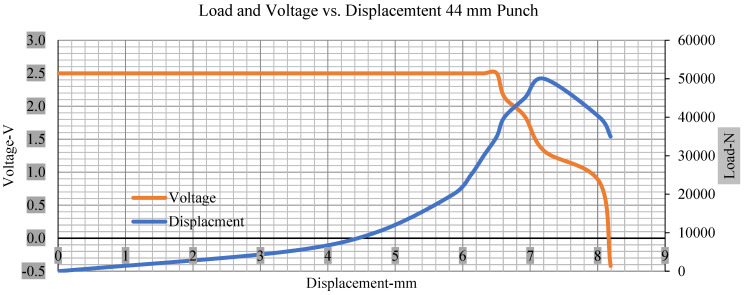
Load and voltage vs. displacement 44 mm punch greater than 10% soc.

For the initial tests of cells 1, 2, and 3, 4, as shown in Figure 5, both load and voltage are graphs of a displacement function. The voltage drops when a short circuit occurs in any of the indentation tests. Figure 6, Figure 7, Figure 8 and Figure 9 show each cell with greater than 10% soc. It is understood that the onset of a short circuit is directly connected with the cell’s mechanical failure. Test data also revealed, with an increase in punch radius, the beginning of electric short circuit successively occurred later, starting at time = 180 s, time = 260 s, time = 289 s, and time = 304 s or small, medium, large, and a flat cylindrical punch sizes, respectively. A hemispherical punch with 44 mm size did not produce any short circuit or drop in voltage until 5 kN, as shown in Figure 9.

**Figure 10:**
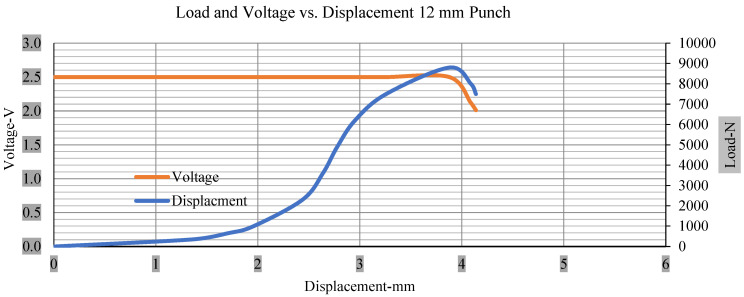
Load and voltage vs. displacement 12 mm punch less than 10% soc.

**Figure 11:**
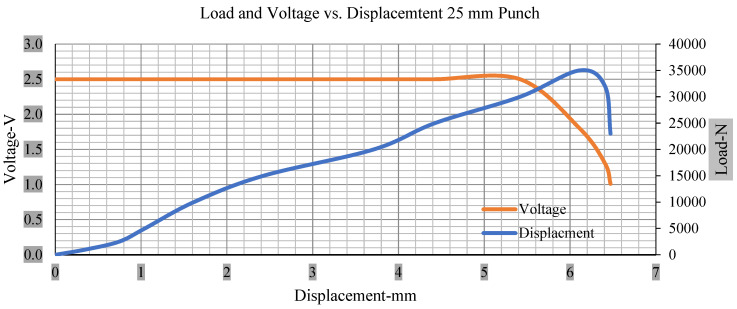
Load and voltage vs. displacement 25 mm punch less than 10% soc.

**Figure 12:**
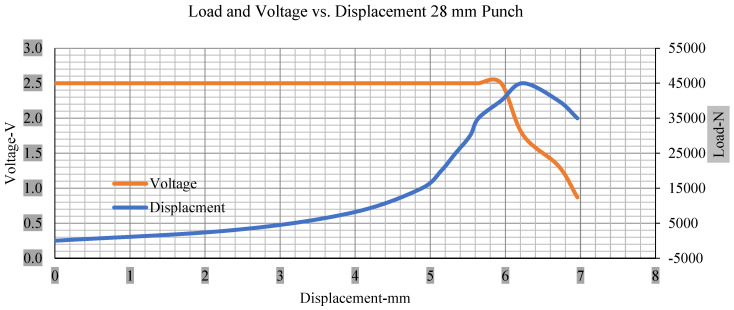
Load and voltage vs. displacement 28 mm punch less than 10% soc.

**Figure 13:**
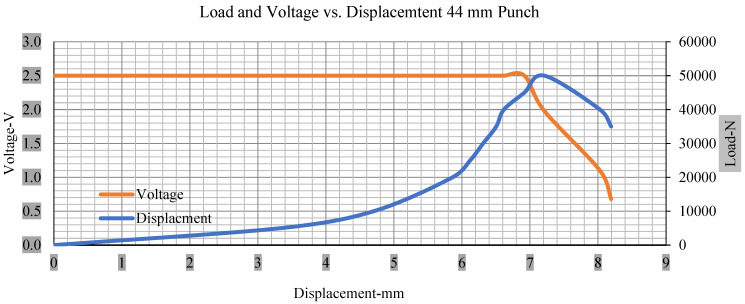
Load and voltage vs. displacement 44 mm punch less than 10% soc.

Figure 6, Figure 7, Figure 8, Figure 9, Figure 10, Figure 11, Figure 12 and Figure 13 shows that the voltage drop is at the point of a short circuit in all tests. It can be determined that the cut-off voltage of all punches starts from 2.5, but the drops in voltage displacement and peak loads are different for both cells for all punch types in both soc conditions. However, a drop-in voltage of small punch with size 12 mm, medium punch with size 28 mm, 44 mm, and flat cylindrical punch with size 25 mm goes on an ending of 1.90, 0.65, −0.42, and 0.75. Nevertheless, a large punch with a size of 44 mm shows −0.42 voltages at 50 kN load when the load is removed back from the cell, as shown in Figure 9 for greater than 10% soc.

Thus, the values for less than 10% soc drop in the voltage of small, medium punch, large punch, and flat cylindrical punch are 1.98, 1.01, 0.81, and 0.68. A drop in voltage rebound is found after a preliminary drop for punch indentation for all cells, and then the voltage progressively lowered to 0 to −0.30 over 15 s.

With the exception of the small and medium punch studies, local deformation that can be securely tolerated from 6 mm (small cell) to 10 mm (medium cell) through the cell levels and even 12 mm punch also did not produce much deformation, as shown in Figure 6, Figure 7, Figure 8, Figure 9, Figure 10, Figure 11, Figure 12 and Figure 13.

As predicted, the cells trigger a voltage drop just under the large punch of 44 mm, as shown in Figure 9 and the displacement graph, and it is considerably deeper and quicker than other punch indentations. The hassle is pushed through the basic geometry of a massive punch that is defined in terms of the tip’s central angle and radius. These findings measure the pattern that is expected in every other situation; those batteries can safely handle extra blunt object intrusion. For 44 mm punch with less than 10% soc, the displacements are 6.474 and 6.324, and, for the other two cells with greater than 10%, the soc displacements are 6.493 and 6.503, respectively, as shown in Figure 17.

This analysis measures the point between two of these similarities and variations. Moreover, an investigation has been performed in relation to how these materials’ properties can be changed when indentation moves from the middle to the corners of the cell. However, in these experiments, the indentation punch position has also been changed from the mid to corner, and the readings are shown for less and greater than 10% soc.

### 3.2. Effect of Electrolyte with the State of Charge

**Figure 14:**
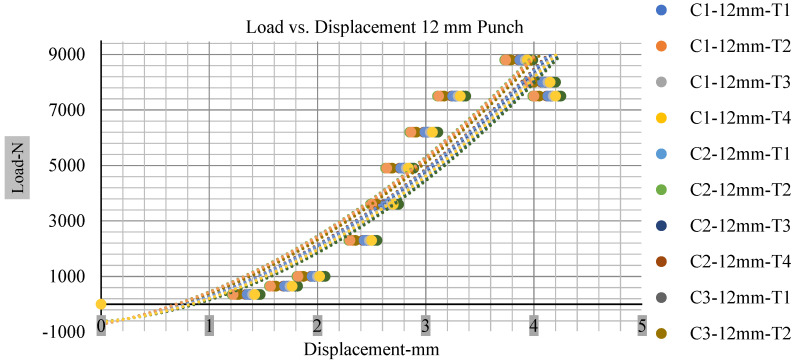
Load vs. displacement 12 mm low to peak loads.

**Figure 15:**
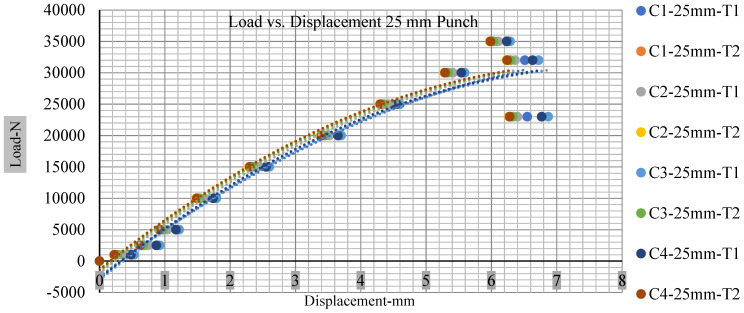
Load vs. displacement 25 mm low to peak loads.

**Figure 16:**
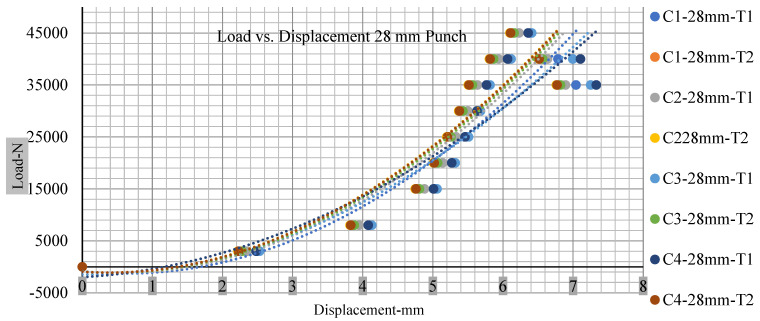
Load vs. displacement 28 mm low to peak loads.

**Figure 17:**
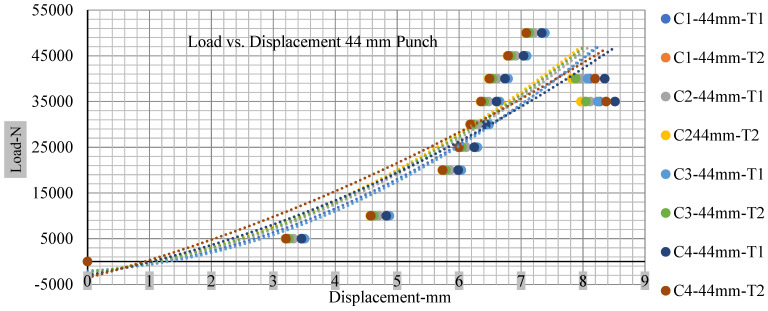
Load vs. displacement 44 mm low to peak loads.

Different indentation tests have been performed on less than 10% state of charge cell to evaluate the electrolyte impact on lithium cells’ loading response. Tests were performed with more than 10% state of charge cell; all of the experiments have been conducted with 12 mm, 25 mm, 28 mm, and 44 mm diameter punch, and all the experiments have been completed at a rate of 1 mm/min. loading. All of the punch tests are performed twice, except for the punch with a 12 mm diameter. These tests have been performed on both soc cells. The cells with less than 10% soc have a much more rigid reaction and a high force level, being significantly more than 10% soc. In contrast, for the estimated force for the last two cells with less than 10% soc, with 28 mm punch, the displacements are 5.466 and 5.316, and, for the first two cells with greater than 10%, the soc displacements are 5.529 and 5.506, as shown in Figure 14, Figure 15, Figure 16 and Figure 17.

The short circuit onset is observed from the voltage decrease in the cell, which has greater than 10% soc; the punch with a 44 mm diameter starts from 2.14 and ends at −0.42, which clearly shows a high short circuit.

Thus, short circuit, punch displacements with greater than 10% SOC at onset are 2.952 mm, 3.712 mm, 5.529 mm, and 6.503 mm, and, similarly for less than 10% soc, 3.88 mm, 5.42 mm, 5.94 mm, and 6.92 mm for a small, medium, large, and a flat cylindrical punch. In less than 10% soc, the flat cylindrical punch has a diameter of 28 mm, in which the short circuit force initiates no drop in.

However, for a hemispherical punch with 44 mm diameter, the onset of the short circuit coincides with a load drop, and the resistance is measured, compression is reported, and a drop in resistance is shown from 900 to 0.

No calculation of the voltage drop is found for the cells with less than 10% soc, which causes a short circuit. However, the same patterns follow the cell’s homogenized reaction and become softer in the wet state. Comparing the onset contrast to a cell with less than 10% soc and loss is earlier when a cell with greater than 10% soc is tested.

### 3.3. Loading Positions with Different State of Charge

This experiment has been conducted to explore the impact of changing load positions on cells. For this purpose, two cells are manufactured with a soc less than 10%, and two cells are more than 10% soc to check the effects of a short circuit; on the other hand, during experiments, all of the cells are kept in two-gallon zip-lock bags [70]. All of these cells are subjected to the punch indentation loads, and variations in the cells’ response have been examined. Tests are performed on lithium-ion cells with pouch cover. However, previous researches have demonstrated that not applying thin foil fused aluminum/polyester of pouch cover, under these loading conditions and not adding power or strength to the cell and calculated data of the cell explicitly represent the electrode/separator stack properties [69].

The hemispherical punch loading has been extended to a pointed matrix, starting from the cell center and moving towards the corners, and then the findings are compared. The quasi-static is tested at a 1 mm/min. rate loading time, checking. Apart from this, the results are consistent with the experiments. For this purpose, indentation testing with four different types of diameters punches has been carried out. Several hemispherical and flat cylindrical punch experiments have been conducted equally on greater than 10% and less than 10% soc cells at different locations. Several punches have been made on four cells. The measurement is placed in the middle of the cell between 30 mm–55 mm from the long edge of the cell. These are punches with a different punch diameter, and the distance is different on all cells, between 50 mm–60 mm from the long edge in the second test, and 10–15 mm from the long edge in the third. A fourth is conducted on the corner of the cell. All of the tests are conducted in this measurement, but the sizes of punch are different, and variability has been established from the multiple experiments.

Figure 5a–d presents the coordinates of the punch. Some tests are in the middle, some are on the corner, and some are between the hub and center to check the maximal measurement of force with greater than 10% soc (with 44 mm punch diameter) regarding a crosshead of 7.21 mm, 50 kN. Some of the tests are performed with a hemispherical punch with a 44 mm diameter on the corner of the cell, and some are performed in the middle of the cell. The center of cell tests has a great deal of lower maximum power, which is predicted from the cell’s edge containing less substance than the middle of the cell.

**Figure 18:**
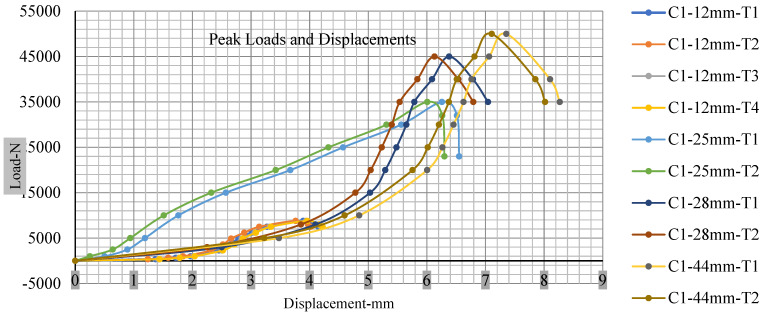
Cell 1 peak loads and displacements.

**Figure 19:**
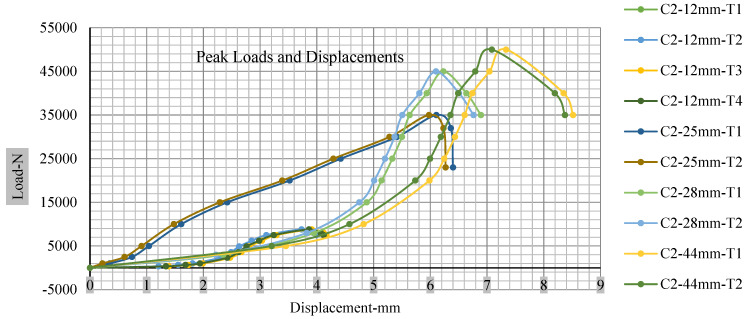
Cell 2 peak loads and displacements.

**Figure 20:**
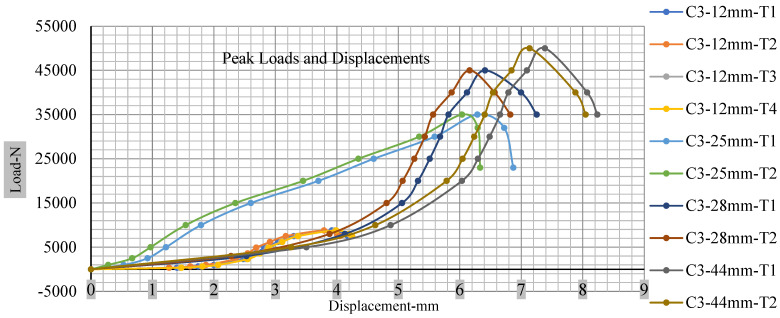
Cell 3 peak loads and displacements.

**Figure 21:**
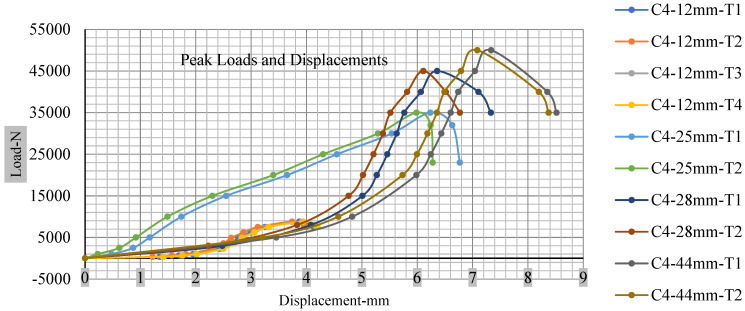
Cell 4 peak loads and displacements.

Figure 18, Figure 19, Figure 20 and Figure 21 show the peak load and deformation depth at short circuit versus all four cells and different punch diameters. It is fascinating to observe that the short circuit’s peak load should almost linearly grow high with an extended punch diameter. In this experiment, a punch diameter of 44 mm creates a large voltage drop with greater than 10% soc, as shown in the graph presented in Figure 9. Nevertheless, the load increases with the increase in the punch’s diameter, as shown in Figure 18, Figure 19, Figure 20 and Figure 21, and high displacements are shown in Figure 14, Figure 15, Figure 16 and Figure 17. The peak loads and displacements differential between less than 10% soc and greater than 10% soc with hemispherical punches are applied. The hemispherical punch with 28 mm diameter shows maximum force measurements of 40–45 kN with lesser and greater than 10% soc. The 44 mm diameter punch shows 50 kN with lesser and greater than 10% soc. Table A1, Table A2, Table A3 and Table A4 (Appendix A) show force-displacement readings.

This allows researchers to use a single cell for running several tests without the fear that the punch’s position would impact the results and only the punch diameter can change it. This study discusses which variables in similar experiments can be carefully regulated and left accessible for operator convenience. Table A1, Table A2, Table A3 and Table A4 (Appendix A) the test readings on each cell.

**Figure 22:**
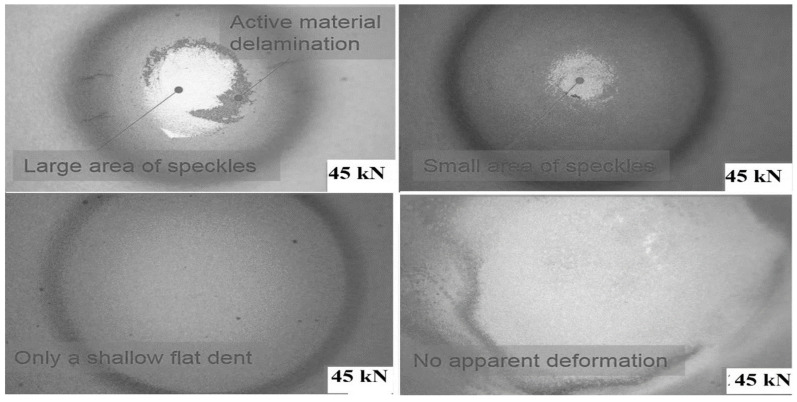
Post-mortem examination of the loaded area.

**Figure 23:**
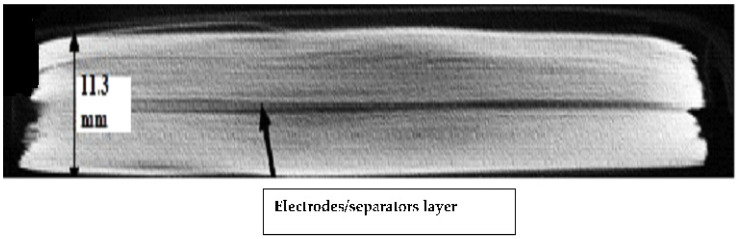
Interior of battery cells after opening the cover of a pouch cell, the first layer seen is a separator layer.

The involvement of electrolytes has been shown to have a direct effect on the cell’s reaction. When a load is applied to the cell, with an increasing load, electrolyte structure also changes because of this internal short circuit that changes the cell’s condition. In all indentation punch filling cases, the pouch cells with an electrolyte with less than 10% soc have soc in all types of diameters. Therefore, researchers should pay particular attention while testing a cell’s mechanical reaction. During the indentation process, the local deformation of each separator before the commencement of the short circuit is another indirect evidence of the fracture sequence. For this reason, separators between each of the four electrode pairs from the pouch cell subjected to 45 kN indentation were investigated using SEM, as shown in Figure 22. The depletion of the thickness separator in the area just below the indenter (center) was compared to the area further out from the indenter (the margin area) as shown in Figure 23.

## 4. Conclusions

This research investigates findings from three related major study areas: material description of Lithium cells used in experiments, the inner short circuit in lithium-ion battery cell starting point when the load is being applied, and the maximum reaction of cell load-carrying capacity. Performance characteristics for the lithium-ion pouch battery are calculated in different conditions.

Four types of hemispherical punch indentation tests have been used to obtain better results to build a computational model pouch-type lithium-ion battery cells. The force-displacement curves and the internal short circuits for these experiments operating at a rate of 1 mm/min. have been very compatible with the results found in this analysis, and they have validated these results.

The overall conclusion is that the cell’s electrical short circuit is not instigated instantaneously when loading begins. These data can feed into numerical models and help to determine nominal battery pack loading conditions for improved staff protection under an optimal cell with optimization conditions. These results will further define the initial requirements in a previously developed successful modeling tool and enable the model to more accurately predict individual cell behavior in crash scenarios.

## Figures and Tables

**Table 1:** Model: GPLFP 11192320ES-50 Ah.

Cell Specifications	Values
Nominal Capacity.	50 Ah
Nominal Voltage	3.2 V
Width	193 mm max
Length	320 mm max
Thickness	11.3 mm max
Weight	1300 g
Tab width	50 ± 0.5 mm
Electrolyte	lithium hexafluorophosphate (LiPF6)
Center	100 ± 2.0 mm

Test cell specifications [72].

**Table 2:** Number of tests on each cell.

Cell No.	Order & Number in Experiments Performed
1	With >10% soc
	Medium (2)
	Large (2)
	Small (4) one voltage
	Cylindrical (2)
2	With >10% soc
	Small (4), one voltage
	Medium (2)
	Large (2)
	Cylindrical (2
3	With <10% soc
	Large (2),
	Medium (2)
	Small (4) one voltage
	Cylindrical (2)
4	With <10% soc
	Cylindrical (2)
	Large (2)
	Medium (2)
	Small (4) one voltage

## Data Availability

Data will be available on suitable demand.

## Appendix A

**Table A1:** Peak force and associated displacement for punch test cell 1 (C1).

12 mm Load (N)	25 mm Load (N)	28 mm Load (N)	44 mm Load (N)	C1-12 mm-T1	C1-12 mm-T2	C1-12 mm-T3	C1-12 mm-T4	C1-25 mm-T1	C1-25 mm-T2	C1-28 mm-T1	C1-28 mm-T2	C1-44 mm-T1	C1-44 mm-T2
0	0	0	0	0.0	0	0	0	0	0	0	0	0	0
350	1000	3000	5000	1.37	1.24	1.42	1.44	0.5	0.25	2.5	2.25	3.475	3.225
650	2500	8000	10,000	1.716	1.586	1.766	1.786	0.89	0.64	4.1	3.85	4.845	4.595
1000	5000	15,000	20,000	1.97	1.84	2.02	2.04	1.19	0.94	5.03	4.78	6.005	5.755
2300	10,000	20,000	25,000	2.45	2.32	2.5	2.52	1.76	1.51	5.29	5.04	6.265	6.015
3600	15,000	25,000	30,000	2.65	2.52	2.7	2.72	2.57	2.32	5.48	5.23	6.455	6.205
4900	20,000	30,000	35,000	2.79	2.66	2.84	2.86	3.67	3.42	5.65	5.4	6.625	6.375
6200	25,000	35,000	40,000	3.013	2.883	3.063	3.083	4.57	4.32	5.786	5.536	6.761	6.511
7500	30,000	40,000	45,000	3.27	3.14	3.32	3.34	5.56	5.31	6.087	5.837	7.062	6.812
8800	35,000	45,000	50,000	3.89	3.76	3.94	3.96	6.256	6.006	6.38	6.13	7.355	7.105
8000	32,000	40,000	40,000	4.1	3.97	4.15	4.17	6.512	6.262	6.786	6.536	8.101	7.851
7500	23,000	35,000	35,000	4.15	4.02	4.2	4.22	6.55	6.3	7.042	6.792	8.267	8.017

**Table A2:** Peak force and associated displacement for punch test cell 2 (C2).

12 mm Load (N)	25 mm Load (N)	28 mm Load (N)	44 mm Load (N)	C2-12 mm-T1	C2-12 mm-T2	C2-12 mm-T3	C2-12 mm-T4	C2-25 mm-T1	C2-25 mm-T2	C2-28 mm-T1	C2-28 mm-T2	C2-44 mm-T1	C2-44 mm-T2
0	0	0	0	0.0	0	0	0	0	0	0	0	0	0
350	1000	3000	5000	1.34	1.21	1.39	1.34	0.35	0.22	2.35	2.22	3.455	3.205
650	2500	8000	10,000	1.686	1.556	1.736	1.686	0.74	0.61	3.95	3.82	4.825	4.575
1000	5000	15,000	20,000	1.94	1.81	1.99	1.94	1.04	0.91	4.88	4.75	5.985	5.735
2300	10,000	20,000	25,000	2.42	2.29	2.47	2.42	1.61	1.48	5.14	5.01	6.245	5.995
3600	15,000	25,000	30,000	2.62	2.49	2.67	2.62	2.42	2.29	5.33	5.2	6.435	6.185
4900	20,000	30,000	35,000	2.76	2.63	2.81	2.76	3.52	3.39	5.5	5.37	6.605	6.355
6200	25,000	35,000	40,000	2.983	2.853	3.033	2.983	4.42	4.29	5.636	5.506	6.741	6.491
7500	30,000	40,000	45,000	3.24	3.11	3.29	3.24	5.41	5.28	5.937	5.807	7.042	6.792
8800	35,000	45,000	50,000	3.86	3.73	3.91	3.86	6.106	5.976	6.23	6.1	7.335	7.085
8000	32,000	40,000	40,000	4.07	3.94	4.12	4.07	6.362	6.232	6.636	6.506	8.351	8.196
7500	23,000	35,000	35,000	4.12	3.99	4.17	4.12	6.4	6.27	6.892	6.762	8.517	8.373

**Table A3:** Peak force and associated displacement for punch test cell 3 (C3).

12 mm Load (N)	25 mm Load (N)	28 mm Load (N)	44 mm Load (N)	C3-12 mm-T1	C3-12 mm-T2	C3-12 mm-T3	C3-12 mm-T4	C3-25 mm-T1	C3-25 mm-T2	C3-28 mm-T1	C3-28 mm-T2	C3-44 mm-T1	C3-44 mm-T2
0	0	0	0	0.0	0	0	0	0	0	0	0	0	0
350	1000	3000	5000	1.4	1.27	1.45	1.47	0.53	0.28	2.53	2.28	3.505	3.255
650	2500	8000	10,000	1.746	1.616	1.796	1.816	0.92	0.67	4.13	3.88	4.875	4.625
1000	5000	15,000	20,000	2	1.87	2.05	2.07	1.22	0.97	5.06	4.81	6.035	5.785
2300	10,000	20,000	25,000	2.48	2.35	2.53	2.55	1.79	1.54	5.32	5.07	6.295	6.045
3600	15,000	25,000	30,000	2.68	2.55	2.73	2.75	2.6	2.35	5.51	5.26	6.485	6.235
4900	20,000	30,000	35,000	2.82	2.69	2.87	2.89	3.7	3.45	5.68	5.43	6.655	6.405
6200	25,000	35,000	40,000	3.043	2.913	3.093	3.113	4.6	4.35	5.816	5.566	6.791	6.541
7500	30,000	40,000	45,000	3.3	3.17	3.35	3.37	5.59	5.34	6.117	5.867	7.092	6.842
8800	35,000	45,000	50,000	3.92	3.79	3.97	3.99	6.286	6.036	6.41	6.16	7.385	7.135
8000	32,000	40,000	40,000	4.13	4	4.18	4.2	6.722	6.292	6.996	6.566	8.071	7.881
7500	23,000	35,000	35,000	4.18	4.05	4.23	4.25	6.87	6.33	7.252	6.822	8.237	8.047

**Table A4:** Peak force and associated displacement for punch test cell 4 (C4).

12 mm Load (N)	25 mm Load (N)	28 mm Load (N)	44 mm Load (N)	C4-12 mm-T1	C4-12 mm-T2	C4-12 mm-T3	C4-12 mm-T4	C4-25 mm-T1	C4-25 mm-T2	C4-28 mm-T1	C4-28 mm-T2	C4-44 mm-T1	C4-44 mm-T2
0	0	0	0	0.0	0	0	0	0	0	0	0	0	0
350	1000	3000	5000	1.35	1.22	1.4	1.42	0.48	0.23	2.48	2.23	3.455	3.205
650	2500	8000	10,000	1.696	1.566	1.746	1.766	0.87	0.62	4.08	3.83	4.825	4.575
1000	5000	15,000	20,000	1.95	1.82	2	2.02	1.17	0.92	5.01	4.76	5.985	5.735
2300	10,000	20,000	25,000	2.43	2.3	2.48	2.5	1.74	1.49	5.27	5.02	6.245	5.995
3600	15,000	25,000	30,000	2.63	2.5	2.68	2.7	2.55	2.3	5.46	5.21	6.435	6.185
4900	20,000	30,000	35,000	2.77	2.64	2.82	2.84	3.65	3.4	5.63	5.38	6.605	6.355
6200	25,000	35,000	40,000	2.993	2.863	3.043	3.063	4.55	4.3	5.766	5.516	6.741	6.491
7500	30,000	40,000	45,000	3.25	3.12	3.3	3.32	5.54	5.29	6.067	5.817	7.042	6.792
8800	35,000	45,000	50,000	3.87	3.74	3.92	3.94	6.236	5.986	6.36	6.11	7.335	7.085
8000	32,000	40,000	40,000	4.08	3.95	4.13	4.15	6.632	6.242	7.106	6.516	8.351	8.196
7500	23,000	35,000	35,000	4.13	4	4.18	4.2	6.77	6.28	7.332	6.772	8.517	8.373

## References

[1] Wang Q., Mao B., Stoliarov S.I., Sun J. (2019). A review of lithium ion battery failure mechanisms and fire prevention strategies. Prog. Energy Combust. Sci..

[2] Feng X., Fang M., He X., Ouyang M., Lu L., Wang H., Zhang M. (2014). Thermal runaway features of large format prismatic lithium ion battery using extended volume accelerating rate calorimetry. J. Power Sources.

[3] Ali M.Y., Lai W.J., Pan J. (2015). Computational models for simulation of a lithium-ion battery module specimen under punch indentation. J. Power Sources.

[4] Ren F., Cox T., Wang H. (2014). Thermal runaway risk evaluation of Li-ion cells using a pinch-torsion test. J. Power Sources.

[5] Zhao C., Sun J., Wang Q. (2020). Thermal runaway hazards investigation on 18650 lithium-ion battery using extended volume accelerating rate calorimeter. J. Energy Storage.

[6] Zhang X., Wierzbicki T. (2015). Characterization of plasticity and fracture of shell casing of lithium-ion cylindrical battery. J. Power Sources.

[7] Zhu J., Wierzbicki T., Li W. (2018). A review of safety-focused mechanical modeling of commercial lithium-ion batteries. J. Power Sources.

[8] Dubaniewicz T.H., Ducarme J.P. (2013). Are Lithium Ion Cells Intrinsically Safe?. IEEE Trans. Ind. Appl..

[9] Luo H., Xia Y., Zhou Q. (2017). Mechanical damage in a lithium-ion pouch cell under indentation loads. J. Power Sources.

[10] Chen Y., Santhanagopalan S., Babu V., Ding Y. (2019). Dynamic mechanical behavior of lithium-ion pouch cells subjected to high-velocity impact. Compos. Struct..

[11] Sarkar A., Shrotriya P., Chandra A. (2019). Modeling of separator failure in lithium-ion pouch cells under compression. J. Power Sources.

[12] Bélanger D. (2018). Editorial Overview: Materials and characterization tools for electrochemical energy storage in batteries and electrochemical capacitors. Mater. Sci..

[13] Zagal J.H., Zagal J.H. (2018). Editorial Overview: Tuning chemistry for better electrocatalysis. Curr. Opin. Electrochem..

[14] Ali L., Nawaz A., Iqbal S., Basheer M.A., Hameed J., Albasher G., Adnan S., Shah R., Bai Y. (2021). Dynamics of Transit Oriented Development, Role of Greenhouse Gases and Urban Environment: A Study for Management and Policy. Sustainability.

[15] An H., Razzaq A., Nawaz A., Noman S.M., Khan S.A.R. (2021). Nexus between green logistic operations and triple bottom line: Evidence from infrastructure-led Chinese outward foreign direct investment in Belt and Road host countries. Environ. Sci. Pollut. Res..

[16] Goodenough J.B., Kim Y. (2010). Challenges for rechargeable Li batteries. Chem. Mater..

[17] Trevey J.E. (2011). Advances and Development of All-Solid-State Lithium-Ion Batteries. Appl. Sci..

[18] Sarkar A. (2017). Thermo-Mechanical Modeling and Parametric Analysis of Lithium-Ion Battery. Master’s Thesis.

[19] Nawaz A., Su X., Nasir I.M. (2021). BIM Adoption and Its Impact on Planning and Scheduling Influencing Mega Plan Projects-(CPEC-) Quantitative Approach. Complexity.

[20] Nawaz A., Waqar A., Shah S.A.R., Sajid M., Khalid M.I. (2019). An innovative framework for risk management in construction projects in developing countries: Evidence from Pakistan. Risks.

[21] Yang Y., Chen H., Zou X., Shi X.-L., Liu W.-D., Feng L., Suo G., Hou X., Ye X., Zhang L. (2020). Flexible carbon-fiber/semimetal Bi nanosheet arrays as separable and recyclable plasmonic photocatalysts and photoelectrocatalysts. ACS Appl. Mater. Interfaces.

[22] Lu H., Zhu Y., Yuan Y., He L., Zheng B., Zheng X., Liu C., Du H. (2021). LiFSI as a functional additive of the fluorinated electrolyte for rechargeable Li-S batteries. J. Mater. Sci. Mater. Electron..

[23] Zhang H., Sun M., Song L., Guo J., Zhang L. (2019). Fate of NaClO and membrane foulants during in-situ cleaning of membrane bioreactors: Combined effect on thermodynamic properties of sludge. Biochem. Eng. J..

[24] Heimes H.H., Kampker A., Lienemann C., Locke M., Offermanns C., Michaelis S., Rahimzei E. (2019). Manufacturing of Lithium-Ion Battery Cell Components.

[25] Zhang L., Wang H., Zhang X., Tang Y. (2021). A Review of Emerging Dual-Ion Batteries: Fundamentals and Recent Advances. Adv. Funct. Mater..

[26] Tan L., Sun Y., Wei C., Tao Y., Tian Y., An Y., Zhang Y., Xiong S., Feng J. (2021). Design of Robust, Lithiophilic, and Flexible Inorganic-Polymer Protective Layer by Separator Engineering Enables Dendrite-Free Lithium Metal Batteries with LiNi0. 8Mn0. 1Co0. 1O2 Cathode. Small.

[27] Tong X., Ou X., Wu N., Wang H., Li J., Tang Y. (2021). High Oxidation Potential≈ 6.0 V of Concentrated Electrolyte toward High-Performance Dual-Ion Battery. Adv. Energy Mater..

[28] Pan Q., Zheng Y., Tong Z., Shi L., Tang Y. (2021). Novel Lamellar Tetrapotassium Pyromellitic Organic for Robust High-Capacity Potassium Storage. Angew. Chemie Int. Ed..

[29] Zhang Y., Liu G., Zhang C., Chi Q., Zhang T., Feng Y., Zhu K., Zhang Y., Chen Q., Cao D. (2020). Low-cost MgFexMn2-xO4 cathode materials for high-performance aqueous rechargeable magnesium-ion batteries. Chem. Eng. J..

[30] Liu Y., Wei Z., Zhong B., Wang H., Xia L., Zhang T., Duan X., Jia D., Zhou Y., Huang X. (2021). O-, N-Coordinated single Mn atoms accelerating polysulfides transformation in lithium-sulfur batteries. Energy Storage Mater..

[31] Minakshi M. (2020). Design, Development and Thermal Analysis of and Stationary Applications. Energies.

[32] Yu D., Mao Y., Gu B., Nojavan S., Jermsittiparsert K., Nasseri M. (2020). A new LQG optimal control strategy applied on a hybrid wind turbine/solid oxide fuel cell/in the presence of the interval uncertainties. Sustain. Energy Grids Netw..

[33] Gong C., Hu Y., Gao J., Wang Y., Yan L. (2019). An improved delay-suppressed sliding-mode observer for sensorless vector-controlled PMSM. IEEE Trans. Ind. Electron..

[34] Kang Y., Zhang Y.-H., Shi Q., Shi H., Xue D., Shi F.-N. (2021). Highly efficient Co3O4/CeO2 heterostructure as anode for lithium-ion batteries. J. Colloid Interface Sci..

[35] Nawaz A., Su X., Din Q.M.U., Khalid M.I., Bilal M., Shah S.A.R. (2020). Identification of the h&s (Health and safety factors) involved in infrastructure projects in developing countries-a sequential mixed method approach of OLMT-project. Int. J. Environ. Res. Public Health.

[36] Commarieu B., Paolella A., Daigle J., Zaghib K. (2018). Toward high lithium conduction in solid polymer and polymer–ceramic batteries. Curr. Opin. Electrochem..

[37] Dai Z., Xie J., Fan X., Ding X., Liu W., Zhou S., Ren X. (2020). Enhanced energy storage properties and stability of Sr (Sc0. 5Nb0. 5) O3 modified 0.65 BaTiO3-0.35 Bi0. 5Na0. 5TiO3 ceramics. Chem. Eng. J..

[38] Ni T., Chang H., Song T., Xu Q., Huang Z., Liang H., Yan A., Wen X. (2019). Non-intrusive online distributed pulse shrinking-based interconnect testing in 2.5 D IC. IEEE Trans. Circuits Syst. II Express Briefs.

[39] Chen C., Wang X., Wang Y., Yang D., Yao F., Zhang W., Wang B., Sewvandi G.A., Yang D., Hu D. (2020). Additive Manufacturing of Piezoelectric Materials. Adv. Funct. Mater..

[40] (2017). Global EV Outlook 2017: Two Million and Counting|en|OECD.

[41] Hao W., Shah S.M.A., Nawaz A., Asad A., Iqbal S., Zahoor H., Maqsoom A. (2020). The Impact of Energy Cooperation and the Role of the One Belt and Road Initiative in Revolutionizing the Geopolitics of Energy among Regional Economic Powers: An Analysis of Infrastructure Development and Project Management. Complexity.

[42] Huo C., Hameed J., Nawaz A., Shah S.A.R., Alqahtani W., Maqsoom A., Anwar M.K. (2021). Scientific Risk Performance Analysis and Development of Disaster Management Framework influencing COVID-19: A Case Study of Developing Asian Countries. J. King Saud Univ..

[43] Ruiz V., Pfrang A., Kriston A., Omar N., Van den Bossche P., Boon-Brett L. (2018). A review of international abuse testing standards and regulations for lithium ion batteries in electric and hybrid electric vehicles. Renew. Sustain. Energy Rev..

[44] Ouyang L., Cao Z., Wang H., Hu R., Zhu M. (2017). Application of dielectric barrier discharge plasma-assisted milling in energy storage materials e A review. J. Alloys Compd..

[45] Chen Y.X. (2016). Simulation-Based Design of Integrated Public Transit and Shared Autonomous Mobility-on-Demand Systems. https://dspace.mit.edu/bitstream/handle/1721.1/117945/1051237118-MIT.pdf?sequence=1&isAllowed=y.

[46] Greve L., Fehrenbach C. (2012). Mechanical testing and macro-mechanical finite element simulation of the deformation, fracture, and short circuit initiation of cylindrical Lithium ion battery cells. J. Power Sources.

[47] Liu B., Yin S., Xu J. (2016). Integrated computation model of lithium-ion battery subject to nail penetration. Appl. Energy.

[48] Goodman J.K.S., Miller J.T., Kreuzer S., Forman J., Wi S., Choi J., Oh B., White K. (2020). Lithium-ion cell response to mechanical abuse: Three-point bend. J. Energy Storage.

[49] Ni T., Yao Y., Chang H., Lu L., Liang H., Yan A., Huang Z., Wen X. (2019). LCHR-TSV: Novel low cost and highly repairable honeycomb-based TSV redundancy architecture for clustered faults. IEEE Trans. Comput. Des. Integr. Circuits Syst..

[50] Liu X., Rao R., Shi J., He J., Zhao Y., Liu J., Du H. (2021). Effect of oxygen vacancy and A-site-deficiency on the dielectric performance of BNT-BT-BST relaxors. J. Alloys Compd..

[51] Zhang H., Guan W., Zhang L., Guan X., Wang S. (2020). Degradation of an Organic Dye by Bisulfite Catalytically Activated with Iron Manganese Oxides: The Role of Superoxide Radicals. ACS Omega.

[52] Yan X., Huang X., Chen Y., Liu Y., Xia L., Zhang T., Lin H., Jia D., Zhong B., Wen G. (2021). A theoretical strategy of pure carbon materials for lightweight and excellent absorption performance. Carbon N. Y..

[53] Sahraei E., Campbell J., Wierzbicki T. (2012). Modeling and short circuit detection of 18650 Li-ion cells under mechanical abuse conditions. J. Power Sources.

[54] Wang H., Kumar A., Simunovic S., Allu S., Kalnaus S., Turner J.A., Helmers J.C., Rules E.T., Winchester C.S., Gorney P. (2017). Progressive mechanical indentation of large-format Li-ion cells. J. Power Sources.

[55] Kurzweil P., Brandt K. (2019). Electrochemical Power Sources: Fundamentals, Systems, and Applications. Chapter-7: Lithium-Secondary Cell: Sources of Risks and Their Effects.

[56] Sun M., Yan L., Zhang L., Song L., Guo J., Zhang H. (2019). New insights into the rapid formation of initial membrane fouling after in-situ cleaning in a membrane bioreactor. Process Biochem..

[57] Lai W., Yusuf M., Pan J. (2014). Mechanical behavior of representative volume elements of lithium-ion battery cells under compressive loading conditions. J. Power Sources.

[58] Wierzbicki T., Sahraei E. (2013). Homogenized mechanical properties for the jellyroll of cylindrical. J. Power Sources.

[59] Spielbauer M., Berg P., Ringat M., Bohlen O., Jossen A. (2019). Experimental study of the impedance behavior of 18650 lithium-ion battery cells under deforming mechanical abuse. J. Energy Storage.

[60] Dixon B., Mason A., Sahraei E. (2018). Effects of electrolyte, loading rate and location of indentation on mechanical integrity of li-ion pouch cells. J. Power Sources.

[61] Berg P., Soellner J., Jossen A. (2019). Structural dynamics of lithium-ion cells—Part I: Method, test bench validation and investigation of lithium-ion pouch cells. J. Energy Storage.

[62] Zhu X., Wang H., Wang X., Gao Y., Allu S., Cakmak E., Wang Z. (2020). Internal short circuit and failure mechanisms of lithium-ion pouch cells under mechanical indentation abuse conditions: An experimental study. J. Power Sources.

[63] Chung S.H., Tancogne-Dejean T., Zhu J., Luo H., Wierzbicki T. (2018). Failure in lithium-ion batteries under transverse indentation loading. J. Power Sources.

[64] Maqsoom A., Babar Z., Shaheen I., Abid M., Kakar M.R., Mandokhail S.J., Nawaz A. (2021). Influence of Construction Risks on Cost Escalation of Highway-Related Projects: Exploring the Moderating Role of Social Sustainability Requirements. Iran. J. Sci. Technol.-Trans. Civ. Eng..

[65] Nawaz A., Su X., Iqbal S., Zahoor H., Asad A., Asghar S., Basit F., Barkat M.Q., Souhail A., Raheel Shah S.A. (2020). Validating a Phenomenological Mathematical Model for Public Health and Safety Interventions Influencing the Evolutionary Stages of Recent Outbreak for Long-Term and Short-Term Domains in Pakistan. Complexity.

[66] Ratner A. (2020). Dynamic Mechanical Compression Impulse of Lithium-Ion Pouch Cells. Energies.

[67] Zhu J., Koch M.M., Lian J., Li W., Wierzbicki T. (2020). Mechanical Deformation of Lithium-Ion Pouch Cells under In-Plane Loads—Part I: Experimental Investigation. J. Electrochem. Soc..

[68] Yoshio M., Brodd R.J., Kozawa A. (2009). Lithium-Ion Batteries: Science and Technologies.

[69] Murata K., Izuchi S., Yoshihisa Y. (2000). Overview of the research and development of solid polymer electrolyte batteries. Electrochim. Acta.

[70] Yong G. (2015). GeePower Energy Technology Co., Limited.: Guangdong, China. https://geebattery.com/about-us.

[71] Sahraei E., Meier J., Wierzbicki T. (2014). Characterizing and modeling mechanical properties and onset of short circuit for three types of lithium-ion pouch cells. J. Power Sources.

[72] Sahraei E., Hill R., Wierzbicki T. (2012). Calibration and finite element simulation of pouch lithium-ion batteries for mechanical integrity. J. Power Sources.

